# Who actually cares for children in slums? Why we need to think, and do, more about paid childcare in urbanizing sub-Saharan Africa

**DOI:** 10.1098/rstb.2020.0430

**Published:** 2021-06-21

**Authors:** Robert C. Hughes, Patricia Kitsao-Wekulo, Ruth Muendo, Sunil S. Bhopal, Elizabeth Kimani-Murage, Zelee Hill, Betty R. Kirkwood

**Affiliations:** ^1^ Department of Population Health, Faculty of Epidemiology and Population Health, London School of Hygiene and Tropical Medicine, Keppel Street, London WC1E 7HT, UK; ^2^ Maternal and Child Wellbeing Unit, African Population and Health Research Center, Nairobi, Kenya; ^3^ Population Health Sciences Institute, Faculty of Medical Sciences, Newcastle University, Newcastle upon Tyne, Tyne and Wear, UK; ^4^ Epidemiology and Public Health, Institute of Global Health, University College London, London, UK

**Keywords:** early childhood development, urban health, child health, childcare, nurturing care

## Abstract

The early years are critical and inform the developmental trajectory of children. This is justifiably attracting growing policy attention. Much of this attention is focused on interventions and policies directed at parents, especially mothers. Yet emerging evidence suggests that increasing numbers of children in rapidly urbanizing low- and middle-income countries are now spending much of their day with other formal and informal childcare providers, including largely unregulated paid childcare providers. This paper summarizes the limited literature about the use of such paid childcare in low- and middle-income countries in sub-Saharan Africa, before considering possible reasons behind the lack of research evidence. Finally, key research gaps and their implications for public health practice are explored, with reference to the ongoing British Academy funded Nairobi Early Childcare in Slums research programme in Nairobi, Kenya. We argue that improving childcare may be an under-explored strategy to help some of the world's most disadvantaged children in the most important period of their lives, and that interventions in this largely informal market should be built on a rigorous research base.

This article is part of the theme issue ‘Multidisciplinary perspectives on social support and maternal–child health’.

## 1. Introduction

The early years—especially the period leading up to a child's third birthday—matter profoundly for the rest of that child's life. This is increasingly well-appreciated, and early childhood development (ECD) is beginning to attract welcome global health policy attention and funding [1].

This growing attention is at least in part due to a growing inter-disciplinary evidence base that emphasizes both the lifelong negative impacts of early adversity [2] and the similarly long-term benefits of effective early intervention [3–5]. In addition, new scientific developments are unpacking the mechanisms through which early adversity, sometimes described as ‘toxic stress’ [6–8], undermines developmental trajectories. This combination means that the case for holistic early childhood intervention has never been stronger. This is particularly so when more narrowly targeted interventions, such as those aiming to improve population nutrition and reduce stunting, are showing less clear benefits on cognitive development than might have been hoped [9].

**Figure 1.**
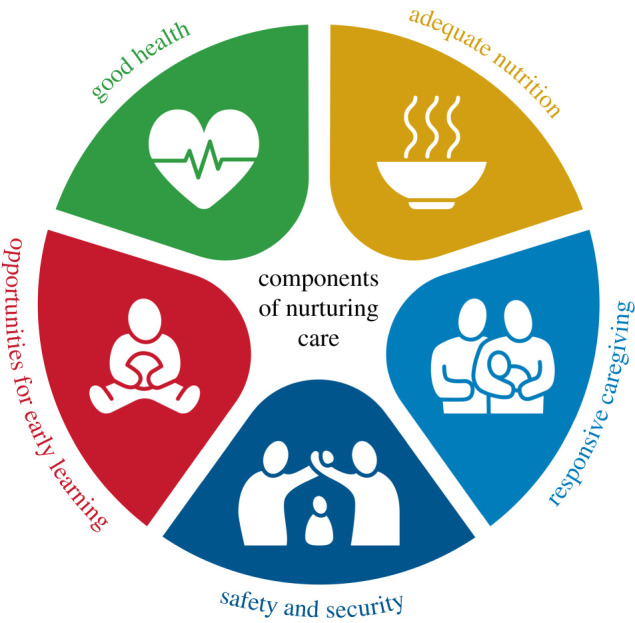
The 2018 WHO/UNICEF Nurturing Care Framework consisting of five domains: early learning, health, nutrition, safety and security, and responsive caregiving (copyright © CC BY-NC-SA 3.0 IGO, World Health Organization 2018). (Online version in colour.)

The inclusion of ECD in major development agendas and global institutions' plans illustrates this growing policy attention. For example, improving ECD is explicitly included within the Global Sustainable Development Goals (SDGs, Goal 4.2) [10] and the World Bank's Human Capital Initiative [11]. Within the global public health discipline, a focus on the early years, and especially the period from zero to three, has been crystallized through the 2018 Nurturing Care Framework [12] and subsequent implementation guidance. This framework was first proposed in the 2016 *Lancet* Early Childhood Development Series [13] and then formally launched at the 2018 World Health Assembly. It emphasizes the universal fundamentals to what constitutes a strong and healthy start to life: security and safety; good health and nutrition; responsive caregiving; and support for early learning (figure 1).

While this recognition of the importance of the early years is welcome, from a practical and policy perspective it is now critical to determine what policies, programmes and interventions can best support babies and toddlers and those who care for them. We are concerned that, through paying insufficient attention to non-parental care providers in general, and paid childcare specifically, ECD strategies are missing a potentially important intervention opportunity in sub-Saharan Africa.

This article seeks to briefly review what we know about paid childcare in sub-Saharan Africa before considering why this issue has received relatively little attention to date. We then go on to consider how through better research and engaging with complex, often informal, childcare systems we might be able to develop a promising holistic early childhood intervention platform.

## 2. A current focus on parental caregivers

The first WHO Guideline addressing ECD, ‘Improving early childhood development’, was published only recently, in 2020 [14]. Implicit in the guideline, and much of the research literature on which it has been developed [14], is a strong, almost universal, focus on primary caregivers (usually parents). Non-parental care providers, especially those who are paid rather than other family members, receive little attention. One, albeit crude illustration of this is the word ‘parent’ appearing 77 times in the 123-page systematic review underpinning the WHO ECD Guideline, compared with zero mentions of any of the words ‘childcare’, ‘daycare’, ‘kindergarten’ or ‘nursery’. Indeed, these terms were not included in the review's search strategy [2].

While parents as primary caregivers clearly play a central role in the upbringing of most children, for many young children (especially those growing up in informal urban areas), other caregivers, including non-family members, play crucial roles too [15,16]. However, it is implicit in much current ECD programming that the mother is the primary, or even the only, person to support children in the early years. This is exemplified by the focus on home visiting [17] or parent group interventions [18]—which often assume that the child is home with the mother [19]. While these parent/home-centred intervention approaches are clearly important, we feel it is important that early childhood interventions are not limited to targeting parents as the focus, and the home as the locus, of intervention.

## 3. What do we know about the use of paid childcare in sub-Saharan Africa?

Africa is rapidly urbanizing [20], mostly to slums, and this is radically changing early childhood care. A number of linked drivers seem to be leading to an increase in the use of childcare of low and unknown quality [21].

As urbanization occurs, family structures change, with a growing tendency towards smaller families [22]. In addition, urban living relies on paid work, whether formal or informal, for most parents, including mothers. This is especially the case for the growing proportion of Africa's urban population who live in slums or informal settlements^1^ [23].

The absence of widespread paid maternity leave and effective social protection systems in urban informal settlements in sub-Saharan Africa means that many working mothers need to return to work soon after childbirth [24]. This is especially the case for the many parents who work in the informal economy, who are likely to have low and irregular earnings, and few if any labour rights [24–26]. In addition, welcome growth in primary and secondary school enrolment means that (at least during school term time) sibling-provided childcare—which itself may not be in the best interests of either the carer or the cared-for child—is less frequently an option for families [15]. Combined with social support networks being limited for some people in urban areas, these factors seem to be driving the increasing need for paid childcare. It is important to also note that alongside these drivers of ‘need’, there appears to be a growing parental demand for childcare in order to provide early learning opportunities and to prepare children for school [27]. Together, these factors—despite user-fees presenting a barrier to access to childcare for many of the poorest families—seem to be driving a growing supply of paid childcare [15].

In sub-Saharan Africa specifically, we are aware of only a handful of studies that have estimated the use of different childcare strategies in the region [15,21,25,28]. These studies suggest that paid childcare—especially in slums—is widely used. The exact form this takes, and the level of formality, vary widely. One recent estimate suggests that 46% of employed and 23% of unemployed parents in the Korogocho slum in Nairobi use paid childcare as the primary childcare strategy [21].

Within the anthropology literature, ‘alloparenting’—defined as caregiving by an individual other than the biological parent—is identified as important in many cultures [29–31]. However, work that has looked at the social networks of young children in African informal settlements to date has not examined who these carers are and how they behave [32].

## 4. Quality of current childcare

There are few studies assessing the quality of current paid childcare provision in sub-Saharan Africa, and it is important to note that there is likely to be considerable heterogeneity across the region. However, the evidence that does exist suggests that the quality of childcare, especially in informal urban areas, is frequently poor across all domains of the Nurturing Care Framework. Staffing ratios are high, training is minimal and learning resources are poor or absent, undermining the potential for early stimulation, responsive care and early learning [33]. Conditions are often unsanitary and unsafe, and the first aid skills of providers have been found to be poor, risking the health and safety of children [34]. Nutrition in informal paid childcare provision has been found to be poor where it has been studied (poor diets, little support provided to even young infants) [35]. Little is known about safeguarding risks in these settings, but numerous reports, including media reports of deaths in care, suggest that systems are absent or weak [36].

This apparently poor quality in many cases reflects the wider context, where children growing up in informal urban areas face numerous adversities [37,38]. But might paid childcare—which brings together some of the world's poorest, most vulnerable children—represent an opportunity for intervention in these most formative years of the life course?

Before considering this in more detail, it is instructive to explore why this issue has, to date, received so little academic or policy attention. In short, why do we know so little about the apparently growing use of paid childcare in sub-Saharan Africa?

## 5. Why has paid childcare been relatively neglected?

First, childcare falls between established lines of accountability. The education sector has a set of policy makers, practitioners and academics, whose attention is focused on school-aged children, and sometimes also one or two pre-school years. Equally, there are many stakeholders working to improve the health system. But who is responsible for childcare in the early years? Is it about early learning (business of the Education Ministry) or child health and development (Ministry of Health territory), or a third, often under-funded, department that deals with women's and children's issues (such as the Ministry of Labour and Social Protection)? The risk of falling between these gaps becomes even more acute when care is largely informal and privately provided, a feature that makes regulation even more challenging.

Second, there remains a question—is childcare the business of the state at all? The role of the state in the care of children is less accepted than involvement in the provision of universal primary education and universal healthcare. This is particularly the case in low- and middle-income countries (LMICs), where the assumption is often—probably incorrectly in many cases—that extended family members are the main or only ‘alloparents’ that need to be considered [29].

Finally, and underlying all of this, there is undeniably a deeply gendered aspect to the neglect of childcare policy and financing [39]. Caring for children in many societies was historically, and still is, seen as women's work. As more women enter the workforce, their role as an earner frequently has to be juggled with other roles, including providing childcare [15]. A disproportionate burden falling on women is a common feature among neglected issues.

Despite the limitations of the current evidence base, the issue of childcare is attracting growing attention. A number of social enterprises and non-governmental organizations (NGOs) are now working to try to improve childcare in LMICs [40,41], and the influential World Bank Early Learning Partnership [42] is starting to explore this agenda.

## 6. Pressing childcare research gaps

Current research gaps risk undermining these efforts. First, it is likely to be challenging to attract and maintain the interest of policy makers and funders without being able to clearly articulate the scale of the issue nor the current quality of childcare provision and the associated equity implications. This is particularly the case in the context of competing demands for attention and resources as the Covid-19 pandemic increases pressure on both domestic and development assistance budgets.

Research seeking to understand the intersectoral inequalities underlying current levels of access to quality childcare (including gender, class, socioeconomic status and in some areas race, ethnic group and migrant status), and that seeks to develop equitable financing approaches (which avoid or minimize out-of-pocket payments among especially the poor) should be central to informing equitable intervention strategies in emerging childcare systems.

In addition, without understanding the beliefs, attitudes and decision-making of the key people involved—most obviously parents/carers but also the often informal and undocumented childcare providers themselves—it will be difficult to design childcare interventions that deliver impact and are scalable, as has been the case with attempts to improve the informal health and education systems [43,44].

Finally, especially in the context of the resource constraints described above, for any intervention strategy to attract attention and funding, research will need to explore the cost-effectiveness of childcare interventions [3]. These analyses should explore not just the short- and long-term benefits to child health, wellbeing, learning and—eventually—earning, but also the co-benefits that access to childcare can bring for parents, especially mothers, and for employers and economies, including if and how improving access to childcare in slums can contribute to female economic empowerment [15,26,45,46].

## 7. Upcoming childcare research in sub-Saharan Africa

In Kenya, our Nairobi Early Childcare in Slums (NECS) Study aims to address some of these gaps [47]. We are exploring the use and quality of paid childcare in a typical Nairobi slum through mixed methods. Interviews and focus groups with parents/carers will explore their decision-making about childcare. Through a household survey, we will estimate the use of paid childcare and other strategies for the care of children under 5, and associations between these strategies and parent, child or household characteristics.

In addition, paid childcare providers in the community will be mapped and their current quality assessed through a combination of a self-report questionnaire and direct observation of practice. Finally, further interviews and focus groups with childcare providers themselves will seek to understand their perspectives and practices [48]. Given that fieldwork for this study has been delayed by the ongoing Covid-19 pandemic, we recently initiated a remote telephone survey sub-study of 600 families whom we are following up over 6 months (NECS-CiT, Covid Impact Tracker) to track how the pandemic, and the associated control measures, are impacting on the care of young children in slums [49].

This work complements other initiatives examining the role of ‘communities of practice’ among childcare providers [50] and the way in which access to improved childcare can contribute to female economic empowerment with likely benefits for the whole household [45].

Through these studies, and linked advocacy, we hope that policy makers and funders will better understand and engage with childcare systems in urbanizing sub-Saharan Africa. In turn, we hope that improving childcare quality and access can become a promising ECD intervention platform to augment ECD interventions focusing on parents and the home.

## 8. Conclusion

In summary, the potential for improved provision of paid childcare to help promote ECD has received little attention to date. This is despite it offering, especially in rapidly and often informally urbanizing sub-Saharan Africa, the potential to reach children suffering from multiple early childhood adversities at the most important time in their lives. The reasons for this issue being neglected are multiple, but include gaps in accountability, gendered perspectives on childcare and a lack of rigorous research into the issue. We hope that through contributing to addressing the latter, we may be able to help lay the foundations for wider efforts to transform how we think, and what we do, about childcare systems in sub-Saharan Africa and beyond.

## Endnote

1 In this article, we have adopted the approach and terminology as used in the *Lancet* series on ‘The health of people who live in slums’, which defines the term ‘slums’ as distinct from ‘informal urban areas’ based on neighbourhood effects, many of which seem relevant to the discussion around childcare (https://www.thelancet.com/series/slum-health). Nevertheless, our experience is that the terms ‘slum’ and ‘informal urban areas’ are often used somewhat interchangeably.

## Data accessibility

This article has no additional data.
